# Draft Genome Sequence of *Cellulomonas* sp. PS-H5, Isolated from Sekinchan Beach in Selangor, Malaysia

**DOI:** 10.1128/MRA.00956-21

**Published:** 2021-10-28

**Authors:** Nurfatini Radzlin, Kheng Oon Low, Kok Jun Liew, Kian Mau Goh, Iffah Izzati Zakaria, Ummirul Mukminin Kahar

**Affiliations:** a Malaysia Genome Institute, National Institutes of Biotechnology Malaysia, Kajang, Selangor, Malaysia; b Department of Biochemsitry, Faculty of Biotechnology and Biomolecular Sciences, Universiti Putra Malaysia, Serdang, Selangor, Malaysia; c Faculty of Science, Universiti Teknologi Malaysia, Skudai, Johor, Malaysia; University of Southern California

## Abstract

*Cellulomonas* sp. PS-H5 was isolated from Sekinchan Beach in Selangor, Malaysia, using an *ex situ* cultivation method. The present work reports a high-quality draft annotated genome sequence of this strain and suggests its potential glycoside hydrolase enzymes for cellulose, hemicellulose, and starch degradations.

## ANNOUNCEMENT

*Cellulomonas* spp. are alkali-tolerant bacteria known to be industrial enzymes producers (1, 2). *Cellulomonas* sp. PS-H5 was isolated using an *ex situ* cultivation method (3) from wet sediment and mud (upper 15-cm layer) of Sekinchan Beach in Selangor, Malaysia (3.5029N, 101.0945E). Genomic DNA was extracted from strain PS-H5 using the Monarch genomic DNA purification kit (New England BioLabs, Ipswich, MA, USA), following the manufacturer’s instructions. Then, the 16S rRNA gene was amplified by PCR using the 27F and 1492R primers (4) and sequenced. Taxonomic identification was performed by comparing the PS-H5 16S rRNA gene to the sequences in the NCBI and the EzBioCloud 16S databases (5). PS-H5 was most closely related (99.51%) to Cellulomonas pakistanensis NCCP-11^T^ (NCBI GenBank accession number AB618146.1). Here, we report the genome sequence of *Cellulomonas* sp. PS-H5 and analyze its potential applications.

*Cellulomonas* sp. PS-H5 was grown on marine agar (Condalab, Madrid, Spain) at 30°C (pH 6.5) for 24 h. PS-H5 genomic DNA was extracted from a single colony of the cells using the standard protocol of the Monarch genomic DNA purification kit. A paired-end library was prepared using the NEBNext Ultra DNA library preparation kit for Illumina (New England BioLabs) according to the manufacturer’s instructions. Sequencing was performed using the NovaSeq 6000 system with 150-bp paired-end reads (Illumina, San Diego, CA, USA). The raw reads were subjected to trimming using Trimmomatic v0.40 (6), *de novo* assembled using SOAPdenovo v2.04 (7), and annotated using the NCBI Prokaryotic Genome Annotation Pipeline (PGAP) v5.20 (8). The annotated genes were assigned functions using eggNOG (Evolutionary Genealogy of Genes: Non-supervised Orthologous Groups) v5.0 (9). Using digital DNA-DNA hybridization (dDDH) via the Genome-to-Genome Distance Calculator v2.1 (10) and the average nucleotide identity (ANI) function in the EzBioCloud server (11), the PS-H5 genome was compared against all 129 available genomes of *Cellulomonas* spp. in the NCBI genome database (September 2021). Genes encoding carbohydrate-active enzymes (CAZymes) present in the genome of strain PS-H5 were detected using dbCAN2 (12). Default parameters were used for all software tools, unless stated otherwise.

The sequence data comprised 1,425,379,800 bases from 4,751,266 paired-end reads. The PS-H5 genome was assembled into 133 contigs with a coverage of 273×. The genome was 4,481,115 bp (*N*_50_, 64,382 bp) long with a G+C content of 75.3%. A total of 4,117 genes were predicted, including 4,019 protein-coding sequences, 58 RNAs, and 40 pseudogenes. Of these, 97.62% (4,019 genes) were linked to the clusters of orthologous group functions. The genome comparison analyses indicated that PS-H5 was closely related to Cellulomonas pakistanensis NCCP-11^T^ (dDDH, 52.0%; ANI, 93.2%). As the values for the dDDH (<70%) (13) and ANI (<96%) (11) were below the corresponding thresholds, strain PS-H5 might be a new species of *Cellulomonas*. The PS-H5 genome encoded 173 CAZymes, including 104 glycoside hydrolases (GHs), 56 glycoside transferases, 8 carbohydrate esterases, and 5 auxiliary activity enzymes. Strain PS-H5 harbors 14 GHs belonging to GH families 1, 13, and 127 (i.e., β-glucosidase, β-galactosidase, endoglucanase, and α-l-arabinofuranosidase) that are important for cellulose and hemicellulose degradation. Additionally, four GH13 enzymes (two α-amylases, an α-glucosidase, and a pullulanase) involved in starch degradation were detected in the PS-H5 genome. To date, none of the pullulanases from *Cellulomonas* spp. have been characterized. Summarily, the *Cellulomonas* sp. PS-H5 genome provides various GH candidates for potential biotechnological applications.

### Data availability.

The whole-genome shotgun sequence of *Cellulomonas* sp. PS-H5 has been deposited in NCBI GenBank under BioProject accession number PRJNA716128, BioSample accession number SAMN18395727, and GenBank accession number JAHVCI000000000. The version described in this paper is the first version, JAHVCI000000000.1. The raw sequencing reads have been deposited in the NCBI Sequence Read Archive (SRA) under accession number SRR15464914. The 16S rRNA gene sequence of *Cellulomonas* sp. PS-H5 has been deposited in NCBI GenBank under accession number MW786713.1.
